# Error in Byline

**DOI:** 10.1001/jamanetworkopen.2026.42187

**Published:** 2026-10-06

In the Original Investigation titled “Oral Anticoagulants in Patients With Atrial Fibrillation and Advanced CKD Not Requiring Dialysis,”^1^ published September 4, 2026, there was an error in the byline. Two authors had incorrect degrees listed in the byline, both of which should have been PhDs. This article has been corrected.^1^
